# Duck Plague Virus Full-Length UL15 Protein Is a Multifunctional Enzyme Which Not Only Possesses Nuclease Activity but Also Exerts ATPase and DNA-Binding Activity

**DOI:** 10.3390/vetsci12100992

**Published:** 2025-10-14

**Authors:** Qiao Yang, Guoying Zhou, Jing Yang, Mingshu Wang, Ying Wu, Bin Tian, Anchun Cheng

**Affiliations:** 1 Research Center of Avian Diseases, College of Veterinary Medicine, Sichuan Agricultural University, Chengdu 611130, China; yangqiao@sicau.edu.cn (Q.Y.); zguoying@stu.sicau.edu.cn (G.Z.); yangjing5@stu.sicau.edu.cn (J.Y.); 14496@sicau.edu.cn (M.W.); wuy@sicau.edu.cn (Y.W.); 14730@sicau.edu.cn (B.T.); 2 Institute of Veterinary Medicine and Immunology, Sichuan Agricultural University, Chengdu 611130, China; 3 Key Laboratory of Animal Disease and Human Health of Sichuan Province, Chengdu 611130, China; 4 Engineering Research Center of Southwest Animal Disease Prevention and Control Technology, Ministry of Education of the People’s Republic of China, Chengdu 611130, China

**Keywords:** duck plague virus, pUL15, nuclease, DNA-binding, ATPase

## Abstract

The genome of the herpesvirus is a linear double-stranded DNA. The viral genome replicates in the host cell to form a concatemeric DNA, which is then cleaved to produce a unit-length genome. This unit-length genome is packaged into procapsid to produce mature virus particles. The terminase large subunit, pUL15, mediates the cleavage and packaging of viral concatemeric genomes. Duck plague virus (DPV) is a member of the α herpesvirus subfamily. Previous studies have demonstrated that the C-terminal region of DPV pUL15 exhibits non-sequence-specific DNA cleavage activity in vitro, but the characteristics of DPV full-length pUL15 remain unclear. In this study, it was determined that the full-length pUL15 exerted non-sequence-specific nuclease activity. Additionally, full-length pUL15 was capable of binding to DNA and hydrolyzing ATP. To analyze the functional domain of DPV pUL15, pUL15 mutants were constructed, expressed, and purified. The results revealed that DNA-binding and ATPase functions of pUL15 were primarily mediated by its N-terminal region, and the nuclease activity was conducted by its C-terminus. The loss of the nuclease activity did not effect on the DNA-binding and ATPase activity. Taken together, this study’s findings demonstrated that DPV pUL15 is a multifunctional enzyme with ATPase, nuclease, and DNA-binding activities. These results will provide important clues for subsequent studies on the function of terminase and the process of viral genome packaging, and provide a foundational basis for the development of broad-spectrum anti-herpesviral drugs targeting the conserved terminase complex, with direct relevance to veterinary medicine.

## Simple Summary

Duck plague virus (DPV), an alphaherpesvirus, utilizes a terminase complex for genome packaging. This study focuses on the large terminase subunit, pUL15, and characterizes its functional domains. The results showed that the full-length pUL15 protein exhibits three key enzymatic activities: it binds and cleaves DNA non-specifically, and hydrolyzes ATP. Further analysis using truncated mutants mapped the DNA-binding and ATPase functions to the N-terminal region, whereas the nuclease activity was localized to the C-terminus. Notably, these functions are independent, as the loss of nuclease activity did not impair the other two. These findings confirm DPV pUL15 as a multifunctional enzyme essential for genome processing and provide a fundamental basis for understanding the mechanism of viral DNA packaging in herpesviruses.

## 1. Introduction

Most large double-stranded DNA (dsDNA) viruses package their genomes into preformed capsids, as observed in herpesviruses and bacteriophages [1,2,3,4,5,6]. During replication, newly synthesized viral DNA forms head-to-tail concatemeric genomes, which must be cleaved into unit-length genomes and packaged into preassembled procapsids to generate mature virions [7,8]. The viral terminase plays a crucial role in this process, facilitating both the cleavage of the concatemeric genome and its translocation into the procapsid [9,10]. In herpesviruses, the terminase complex typically consists of three subunits: the large subunit pUL15, the small subunit pUL28, and the third subunit pUL33, or their respective homologs [11,12,13,14]. Following assembly in the host cytoplasm, the terminase complex translocates into the host nucleus to perform its function [15]. The large subunit of the terminase complex, pUL15, and its homologs contain two enzymatically active domains: an N-terminus ATPase domain and a C-terminal nuclease domain [16,17]. When pUL15 is null, the cleavage of the viral genome is inhibited and the mature capsid containing DNA cannot be produced; thus, the production of progeny virions is affected [18].

During herpesvirus packaging, the terminase complex docks onto the unique portal vertex, which consists of dodecameric pUL6 in HSV-1. Dodecameric pUL6 forms a circular channel, facilitating the entry of DNA into the preformed capsid in an ATP-dependent manner [19,20,21]. Once the capsid is filled with a unit-length genome, the large terminase subunit executes a second cleavage event, completing the genome packaging process [22]. In addition to the terminase complex and portal protein, several other essential proteins, including UL17, UL25, and UL32, are required for the concatemeric genome cleavage and packaging. Loss of function in any of these proteins results in packaging failure and the accumulation of immature capsids within the nucleus [23,24,25,26].

Despite the low sequence similarity, pUL15 and its homologs are structurally conserved across herpesviruses, underscoring their critical role in genome packaging. Sequence alignment and structure analyses indicate that pUL15 and its homologs belong to the Additional Strand Catalytic E (ASCE) superfamily, with highly conserved ATPase regions containing canonical Walker A and Walker B motifs [27,28,29]. Moreover, the confirmed nuclease-active sites of HSV-1 pUL15, amino acids D509, E581, D706, and D707, are highly conserved among herpesviruses [30]. To date, most studies on herpesvirus pUL15 and its homologs have focused on their nuclease activity. In vitro assays have demonstrated that HSV-1 pUL15 cleaves DNA nonspecifically, as observed in human cytomegalovirus (HCMV) and duck plague virus (DPV) [30,31,32,33]. While some reports have confirmed that pUL28 or its homologs can bind to DNA [34,35,36,37,38], the DNA-binding capability of pUL15 or its homologs remains largely unexplored, leaving uncertainty about their specificity in DNA binding.

The replication of DPV, also named Anatid herpesvirus-1, necessitates the terminase-mediated cleavage of the concatemeric genome to produce mature virions [39]. Sequence alignment reveals that DPV pUL15 is homologous to the large terminase subunits in HSV-1, HCMV, Epstein–Barr virus (EBV), and other herpesviruses, sharing highly conserved nuclease activity sites and Walker motifs. Previous research has demonstrated that the C-terminus domain of DPV pUL15 only possesses non-sequence-specific nuclease activity but lacks DNA-binding ability [33]. This study further explored the function of DPV full-length pUL15, revealing that it is a multifunctional enzyme possessing nuclease, ATPase, and DNA-binding activities. It was demonstrated that the N-terminus domain of pUL15 is responsible for ATPase and DNA-binding activities, while the C-terminus domain plays a key role in DNA cleavage.

## 2. Materials and Methods

### 2.1. Cloning, Mutation, and Protein Purification

The DPV full-length UL15 gene sequence (Gene ID: 8223370) was codon-optimized and inserted into the pET28a vector, and then transformed into *E. coli* BL21 for expression. UL15 N-terminus sequence (1 bp to 1428 bp) was amplified using the primers listed in Table A1, and the purified PCR product was cloned into the pET28a vector. A previously constructed plasmid, pET28a-UL15C NM, harboring mutations in the nuclease functional sites (D514A/E586A/D709A/D710A) of the pUL15C-terminal domain, was used as a template to generate a full-length nuclease functional site mutant plasmid (pET28a-UL15NM) via fusion PCR, and then transformed into *E. coli* BL21. Protein expression was performed as described previously [33]. Induced cultures were lysed by sonication, followed by centrifugation to remove debris. The supernatant was incubated with Ni-NTA agarose beads (Smart-Lifesciences, Changzhou, China) at room temperature for 30 min. The beads were washed with wash buffer (50 mM Tris–HCl, 0.5% Triton, 1 M NaCl, and 40–100 mM imidazole, adjusted for different mutant proteins) to remove impurities, and the target proteins were eluted using elution buffer (50 mM Tris–HCl, 250 mM NaCl, and 300 mM imidazole). Protein concentration was measured using a BCA assay kit (Solarbio, Beijing, China) and stored at −80 °C.

### 2.2. Nuclease Activity Assay

The nuclease activity assay was carried out according to the method previously described with some modifications [33]. Purified full-length or mutant pUL15 was incubated with 500 ng of circular pUC118 plasmid or pUC118-CTJ plasmid in nuclease buffer (10 mM Tris–HCl [pH 7.5], 10 mM MgCl_2_·6H_2_O, 1 mM DTT, 50 mM NaCl) in a 20 µL reaction system at 37 °C for 20 min. Hind III (Takara, Beijing, China) digestion of plasmid DNA under identical conditions served as a control to obtain linear plasmid bands. To evaluate the effect of metal ions on the nuclease activity of pUL15FL, 0.5 µM protein was incubated with pUC118 plasmid in nuclease buffer containing 1 mM or 10 mM MgCl_2_, MnCl_2_, or CaCl_2_. Reactions were terminated by adding 6× DNA loading buffer (Beyotime, Shanghai, China) and analyzed using 0.7% agarose gel electrophoresis, followed by ethidium bromide staining.

### 2.3. DNA Binding Assay

The key *cis*-acting signal for herpesvirus genome packaging is the “*a*” sequence, typically containing the conserved motifs Pac1 and Pac2 necessary for DNA cleavage and packaging. A 139 bp dsDNA fragment containing the DPV genome Pac motifs (PAC) or a 139 bp nonspecific sequence (unPAC) was used as DNA-binding substrate. The two fragments were amplified by PCR using the primers shown in Table A1, and the sequences of the two fragments are shown in Table A2. The DNA-binding reaction buffer contained 100 mM Tris–HCl, 0.5 mM Na_2_EDTA, 1 M KCl, 10 mM MgCl_2_, 10 mM DTT, and 25% glycerol. The purified pUL15 or its mutants were incubated with dsDNA Pac and unPac fragments in 20 µL DNA-binding reaction systems at 37 °C for 20 min. Single-stranded DNA (ssDNA) substrates (Invitrogen, Carlsbad, CA, USA) were substituted for dsDNA under similar conditions to evaluate the ssDNA-binding of pUL15FL. The reactions were terminated by adding 6× DNA loading buffer and analyzed on 1% agarose gels with ethidium bromide staining.

### 2.4. ATPase Activity Assay

ATPase activity was measured at 37 °C in reaction buffer containing 1 mM ATP, 40 mM Tris–HCl (pH 7.5), 50 mM NaCl, 2 mM MgCl_2_·6H_2_O, and indicated concentrations of full-length or mutant pUL15. ATP hydrolysis was detected using the Malachite Green Phosphate Detection Kit (Beyotime, Shanghai, China). Pi produced by ATP hydrolysis reacted with malachite green and molybdate to form a green phosphomolybdate complex, which could be detected at 630 nm. To evaluate the hydrolysis of different adenosine nucleotides, 1 mM ATP was replaced with 1 mM ADP as the substrate.

### 2.5. Structure Prediction and Analysis of DPV pUL15

The AlphaFold3 server was utilized (https://alphafoldserver.com/ (accessed on 11 October 2025)) to predict the structure of the monomer and hexamer of DPV pUL15. The sequences submitted to the server corresponded to the Genebank (UL15: YP_003084405.1). The predicted structural models (in PDB format) generated by AlphaFold3 were downloaded for further analysis. All structural visualization, analysis, and figure generation were performed using PyMOL 3.0.3.

## 3. Results

### 3.1. DPV Full-Length pUL15 Exhibits Non-Specific Nuclease Activity, Enhanced by Metal Ions

**Figure 1:**
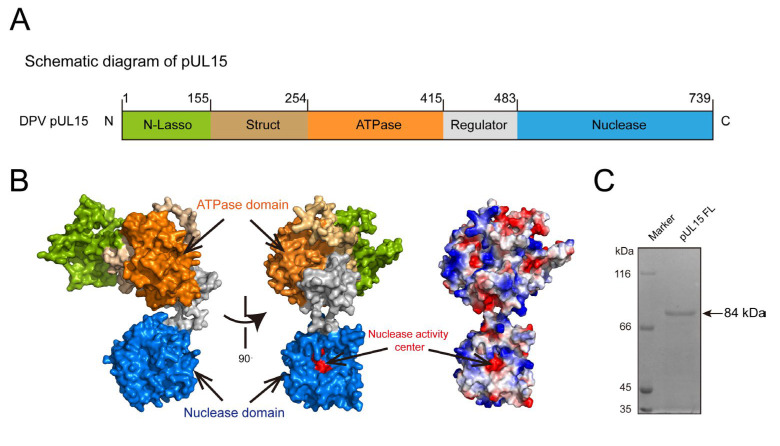
Functional analysis and protein purification of DPV pUL15. (**A**) Schematic diagram of each domain division of full-length pUL15. N-lasso (residues 1 to 155), lassoing the pUL28; the struct domain (residues 156 to 254) fixes the “backbone” of the ATPase domain (residues 255 to 415) by forming an inter-domain four-helix bundle; the regulator domain (residues 416 to 483) connects the ATPase and Nuclease domains (residues 484 to 739). (**B**) Overall structure of pUL15 is predicted by AlphaFold 3. The color scheme is the same as for Figure 1A, and for the surface charge distribution of pUL15, the positive potential is blue, and the negative potential is red. (**C**) SDS-PAGE displaying the purified DPV pUL15FL, which is stained by Coomassie blue (for original figures, see the Supplementary Materials).

A previous study demonstrated that the DPV pUL15C-terminus domain possesses a negatively charged nuclease activity center and exhibits non-specific nuclease activity similar to its homologs in other herpesviruses [33]. Sequence alignment between DPV and HSV-1 pUL15 revealed that DPV full-length pUL15 (pUL15FL) not only contains the nuclease domain, but also harbors the ATPase domain and other conserved domains (Figure 1A,B). To determine whether pUL15FL exhibits specific nuclease activity, the UL15 gene was cloned into the pET28a vector, and then the recombinant His-tagged pUL15 (84 kDa) was expressed and purified (Figure 1C).

**Figure 2:**
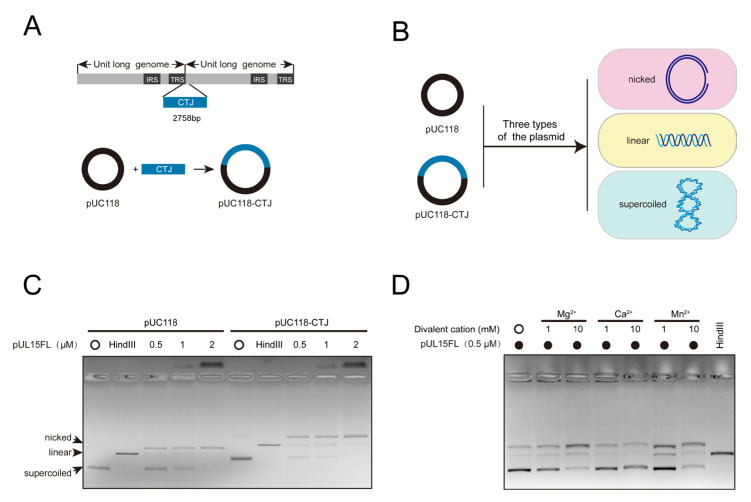
Metal ions can enhance the nuclease activity of full-length pUL15 (for original figures, see the Supplementary Materials). (**A**) Different substrates for the detection of pUL15FL nuclease activity. pUC118 is a vector plasmid, and pUC118-CTJ contains the DPV genome concatemeric terminus junction (CTJ). (**B**) Three forms of plasmid: nicked, linearized, and supercoiled plasmid. (**C**) The nuclease substrate specificity of the pUL15FL was detected with the plasmids pUC118 and pUC118-CTJ. (**D**) The effect of divalent metal ions on the nuclease activity of pUL15FL was detected. The concentration of each metal ion was 1 mM and 10 mM, respectively.

The plasmids pUC118 and pUC118-CTJ, with the latter containing a 2758 bp of concatemeric terminus junction (CTJ), were used as the substrates to assess nuclease activity (Figure 2A). Plasmids commonly contain three types: nicked (open circle), linear, and supercoiled (closed circle) (Figure 2B). Their migration rates in gel electrophoresis from slow to fast are open circle, linear, and supercoiled. The results indicated that the amount of both supercoiled plasmids decreased with increasing protein concentration, but the nicked and linear plasmids increased compared with the control, confirming that pUL15FL still non-specifically cuts DNA even in its full-length state. Interestingly, as the protein concentration increased, the DNA retention near the loading well also increased, suggesting that pUL15FL possesses a DNA-binding ability (Figure 2C).

To investigate the influence of metal ions on the nuclease activity of DPV pUL15FL, Mg^2+^, Ca^2+^, and Mn^2+^ were introduced into the reaction system. To minimize potential interference from DNA binding, a low concentration of pUL15FL was used. As expected, divalent cations enhanced the nuclease activity of pUL15FL, with Mg^2+^ and Mn^2+^ exhibiting stronger effects. Notably, no DNA retention was observed near the loading well under these conditions, indicating that metal ions may not affect the DNA binding ability of pUL15FL (Figure 2D).

### 3.2. DPV pUL15FL Exhibits Non-Specific DNA-Binding Ability

**Figure 3:**
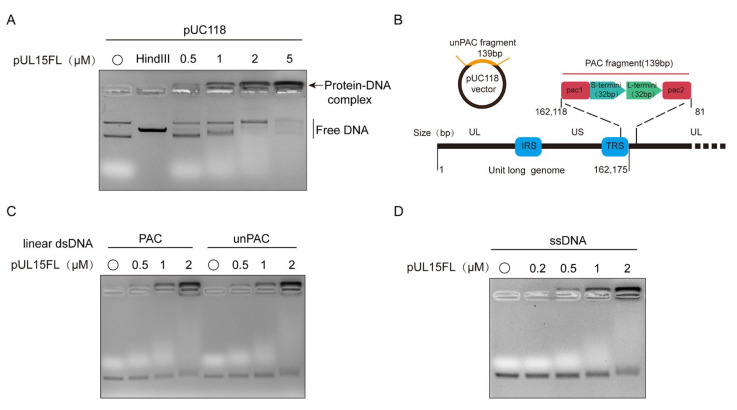
Detection of the DNA-binding ability of pUL15FL (for original figures, see the Supplementary Materials). (**A**) The binding of pUL15FL to plasmid pUC118. Protein–DNA complex and free DNA are indicated with arrows and vertical lines, respectively. (**B**) Schematic diagram of PAC and unPAC fragments. PAC is the sequence of the DPV genome terminus, with a length of 139 bp, and unPAC is a sequence from the vector pUC118 with the same length as PAC. Binding of pUL15FL to dsDNA, both obtained by PCR amplification. (**C**) Binding of pUL15FL to PAC and unPAC. (**D**) The pUL15FL binds to ssDNA.

The above results demonstrated that DPV pUL15FL not only exerted nuclease activity, but also possessed non-specific DNA-binding ability in the nuclease reaction buffer. To determine whether this persisted in a DNA-binding reaction buffer, the pUC118 plasmid was used as substrate. As shown in Figure 3A, as the pUL15FL concentration increased, the free DNA decreased and the DNA remaining near the loading well increased, suggesting that pUL15FL could bind DNA, and the buffer conditions did not affect DNA-binding specificity.

**Figure 4:**
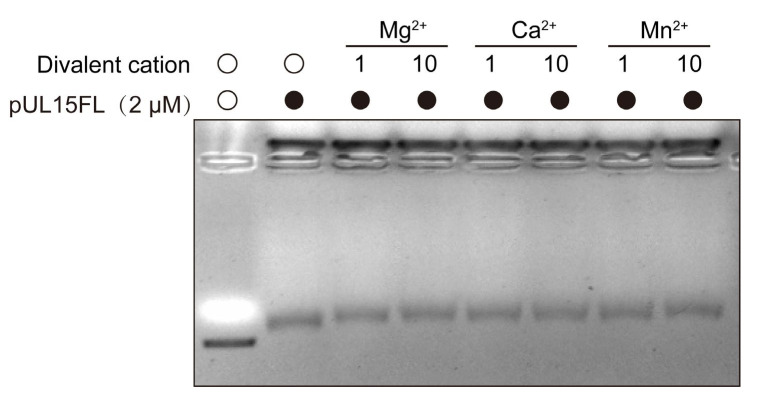
The ability of pUL15FL to bind to DNA does not depend on metal ions (for original figures, see the Supplementary Materials). The effect of different divalent metal ions on the DNA-binding ability of pUL15FL is detected, and the concentration of each metal ion is 1 mM and 10 mM, respectively.

To assess whether pUL15FL can bind to different forms of DNA, linear dsDNA and ssDNA were tested as substrates. The results showed that pUL15FL could bind non-specifically to both dsDNA and ssDNA, indicating that its DNA interaction is independent of a specific form and sequence (Figure 3C,D). Since divalent metal ions could promote the nuclease activity of pUL15, we also wanted to explore whether metal ions could also enhance the binding ability of pUL15FL to DNA. Here, 1 and 10 mM Mg^2+^, Ca^2+^, and Mn^2+^ were used for detection. It was found that, unlike nuclease activity, even in high concentrations, metal ions still could not enhance the DNA-binding ability of pUL15FL. The result showed the DNA-binding capacity of pUL15FL did not depend on metal ions (Figure 4). The above results indicated that pUL15FL could bind to non-specific DNA independently of metal ions.

### 3.3. The N-Terminus of pUL15 Is Responsible for Its Interaction with DNA

**Figure 5:**
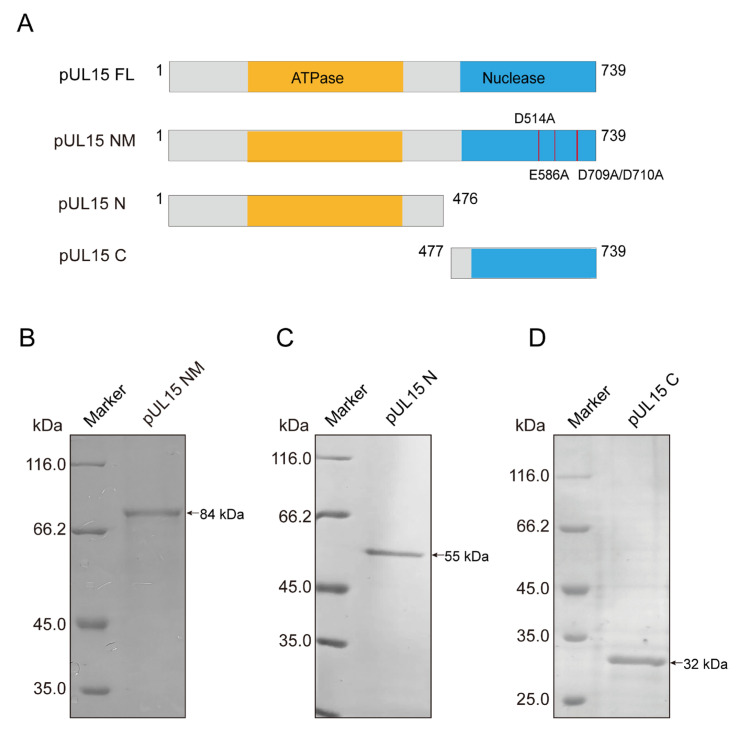
Purification of pUL15 mutants. (**A**) Schematic diagram of UL15FL, UL15NM, UL15N, and UL15C. (**B**–**D**) The purified soluble pUL15NM, pUL15N, and pUL15C are detected by SDS-PAGE and visualized by Coomassie blue staining (for original figures, see the Supplementary Materials).

A previous study has shown that the C-terminal domain of DPV pUL15 exhibits nuclease activity but lacks DNA-binding ability. Combined with the findings of this study, we hypothesized that DNA-binding activity resides in the N-terminal domain. To test this, a plasmid expressing the N-terminus domain of pUL15 was constructed in this study, and the pUL15C, which expressed the C-terminus domain, was constructed in the previous study. Additionally, a nuclease-deficient mutant pUL15NM was generated to examine whether DNA binding depends on its nuclease activity (Figure 5A). Finally, the soluble pUL15NM (84 kDa), pUL15N (55 kDa), and pUL15C (32 kDa) were purified and detected through SDS-PAGE, respectively (Figure 5B–D).

**Figure 6:**
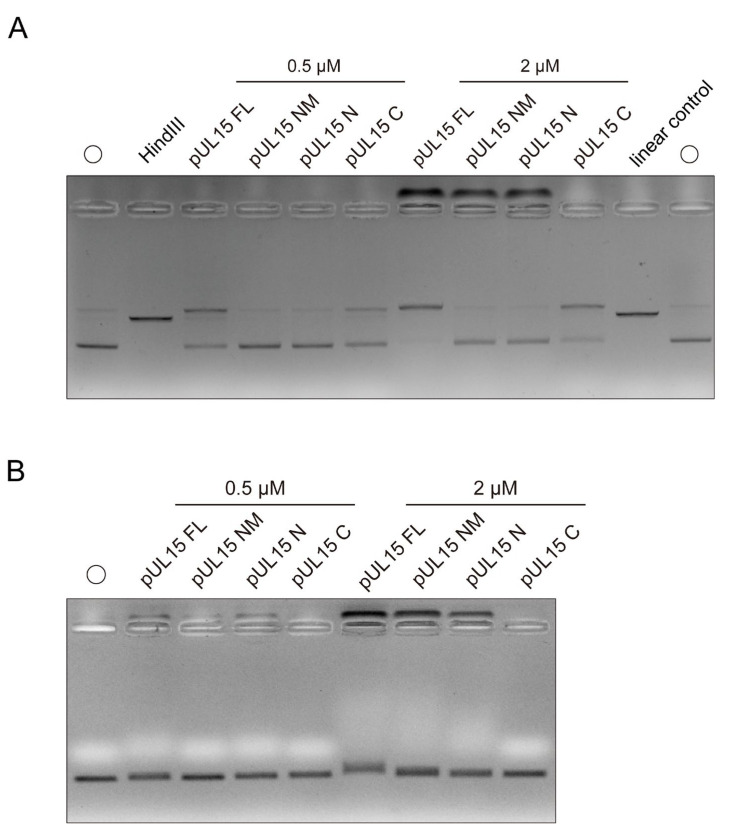
DPV pUL15 N-terminus exerts DNA-binding activity (for original figures, see the Supplementary Materials). (**A**) Detection of the nuclease activity of pUL15 mutants. Plasmid pUC118 is used as the substrate, and the concentration of each protein is 0.5 μM and 2 μM. (**B**) Detection of the DNA-binding ability of pUL15 mutants. The dsDNA, PAC, is used as the substrate, and the concentration of each protein is 0.5 μM and 2 μM.

As shown in Figure 6A, plasmid pUC118 was used as the substrate to detect the nuclease activity in different mutants. Whether under conditions of low concentrations or high concentrations of pUL15FL and its mutants, it was obvious that the nicked plasmid increased but the supercoiled plasmid decreased when only pUL15FL or pUL15C was present in the reaction, compared to the control. This indicated the plasmid was cleaved by pUL15FL and pUL15C, but not pUL15NM and pUL15N (Figure 6A). When the concentration of protein was increased to 2 µM, DNA binding was easier to detect. It was obvious that plasmid DNA accumulated near the loading wells when pUL15FL, pUL15NM, and pUL15N were present in the reaction (Figure 6A). The formation of protein–DNA complex indicated that, in addition to the pUL15 C, the pUL15FL, pUL15NM, and pUL15N all possessed the ability to bind DNA. These results suggested that the N-terminus domain of pUL15 exerted DNA-binding activity, and the loss of nuclease activity did not affect the binding of pUL15 to DNA.

To further rule out the influence of nuclease activity on the observation of pUL15’s ability to bind to DNA, the substrate was replaced by the linear dsDNA, PAC. In Figure 6B, pUL15FL and its mutants, except pUL15C, exhibited obvious DNA-binding activity at high protein concentrations. These findings indicated that the N-terminus region of DPV pUL15 was only capable of DNA binding capacity, while the C-terminus region could only exert nuclease activity. In addition, the loss of nuclease activity of the full-length pUL15 did not affect its DNA-binding capacity.

### 3.4. The N-Terminus of DPV pUL15 Not Only Exerted the Ability to Bind DNA, but Also Possessed ATPase Activity

**Figure 7:**
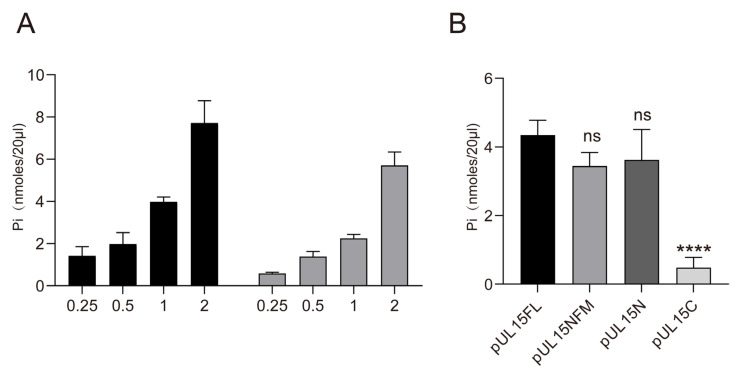
Detection of ATPase activity. (**A**) The hydrolysis of ATP and ADP by pUL15FL is detected. (**B**) The ATPase activity of pUL15 mutants is detected by using ATP as the substrate. The concentration of each protein is 2 μM. Data are mean ± S.D. from at least three biological replicates. One-way ANOVA was performed by using GraphPad Prism software version 9, (ns) *p* > 0.05, (****) *p* < 0.0001.

The ATPase activity of the terminase large subunit has been well characterized in bacteriophages, including SPP1, Sf6, and Phi29 [5,40,41]. In HSV-1, a previous study demonstrated that the pUL15 contains the ATPase domains and hydrolyzes ATP. However, many aspects of pUL15 ATPase function remain unclear. In this study, we found that DPV pUL15FL hydrolyzes both ATP and ADP, with ATP yielding a higher release of phosphate groups than ADP (Figure 7A). Based on the analysis of sequence and structure, it was known that the ATPase region is located at the N-terminus. However, we still did not know whether ATPase activity could be exerted completely without the presence of the C-terminus. Therefore, we detected the ATPase activity of different mutants of DPV pUL15. As shown in Figure 7B, the pUL15N exhibited ATPase activity like pUL15FL, whereas pUL15C did not. Notably, similar to its DNA-binding ability, pUL15NM retained ATPase activity even in the absence of its nuclease function. These findings suggest that the DNA-binding and ATP-hydrolysis activities of pUL15 are independent of its nuclease domain, with the N-terminus playing a crucial role in both functions.

### 3.5. Structure Prediction and Functional Model of DPV pUL15

**Figure 8:**
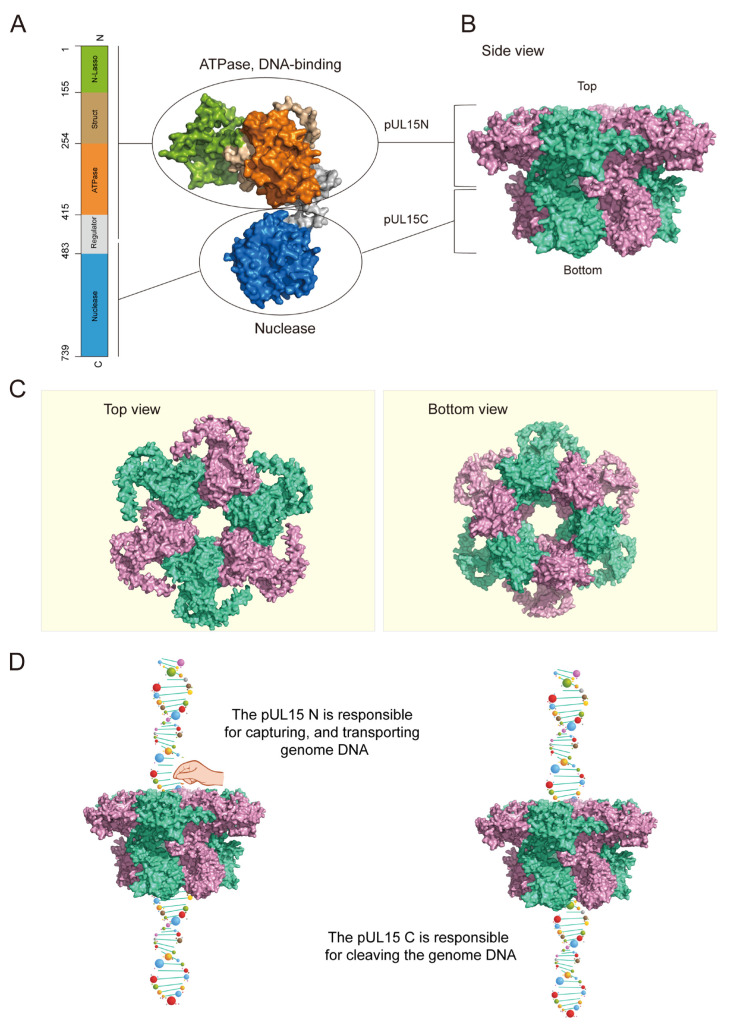
Structure prediction of DPV pUL15 hexamer and the functional model. (**A**) Schematic diagram of DPV pUL15 monomer. (**B**,**C**) Models for DPV pUL15 hexamer. (**B**) Side view of pUL15. (**C**) The left panel is the top view of pUL15, and the right panel is the bottom view of pUL15. (**D**) Functional model of DPV pUL15. The left panel represents the function of the pUL15 N-terminus. pUL15 polymer binds DNA through its channel in the middle of the hexamer, and the N-terminus is responsible for capturing and transporting genomic DNA. The right panel represents the function of the pUL15 C-terminus, which is responsible for cleaving the genomic DNA after the complete genome is packaged.

The above results showed that DPV pUL15 was a multifunctional protein, which simultaneously possessed ATPase, DNA-binding, and nuclease activity. In this study, it was demonstrated that the N-terminus of DPV pUL15 could bind to DNA and could hydrolyze ATP, and the C-terminus of DPV pUL15 possessed the nuclease activity. The structure of the monomer and hexamer of DPV pUL15 was predicted by AlphaFold 3. The prediction results showed that the pUL15 was in an inverted L shape (Figure 8A). The N-terminus domain with DNA binding ability and ATPase activity was located in the upper part of the inverted L shape, while the C-terminus domain with nuclease activity was located in the lower part. The N-terminus domain and the C-terminus domain were connected by a region defined as a regulator (Figure 8A). Based on the composition of the terminase in HSV-1, we predicted the structure of pUL15 hexamer in DPV. The results showed that the DPV pUL15 hexamer presented an inverted cap structure with a channel in the middle (Figure 8B,C). The channel in the middle of the hexamer could be used to bind and transport DNA. According to the confirmed functions and structures of DPV pUL15, we proposed a hypothetical functional model of DPV pUL15 (Figure 8D). During the process of genome package and cleavage in DPV, pUL15 forms a hexamer, and the genome DNA binds to the channel in the hexamer. The pUL15 provides energy for the translocation of DNA in the channel by hydrolyzing ATP, thus allowing the genome to enter the capsid. When the complete genome enters the capsid, the N-terminus of DPV pUL15 releases the genomic DNA, and the C-terminus cleaves the genomic DNA through its nuclease activity, causing the remaining tandem genome to be detached from the capsid.

## 4. Discussion

Herpesviruses possess a linear double-stranded DNA (dsDNA) genome enclosed within an icosahedral capsid [42]. The newly synthesized viral genome exists in host cells as a head-to-tail concatemeric DNA, which must be cleaved into unit-length genomes before packaging into the capsid. This process is mediated by a virus-encoded terminase complex [43,44]. In HSV-1, pUL15, pUL28, and pUL33 form a complex in host cells, which is transported into the nucleus via the nuclear localization signal in pUL15 [11]. The large subunit pUL15 comprises two structural domains: a C-terminal nuclease domain, structurally similar to RNase H, and an N-terminal ATPase domain belonging to the ASCE superfamily [45,46,47]. pUL15 plays a crucial role in cleaving concatemeric genomes and hydrolyzing ATP to generate the energy required for genome translocation [41]. DPV, a member of the herpesvirus family, encodes homologs of HSV-1 terminase subunits, including pUL15, pUL28, and pUL33. It has been shown that DPV pUL15 exhibits DNA cleavage activity, but the nuclease activity is non-sequence-specific in vitro [33]. Similar non-sequence-specific nuclease activity has been reported for the large subunits of terminases in other herpesviruses and bacteriophages [5,30,32,48]. However, herpesvirus concatemeric DNA cleavage in vivo is highly specific. Furthermore, previous studies on the nuclease activity of pUL15 have primarily focused on its C-terminal region. Therefore, the present study aimed to investigate whether full-length DPV pUL15 specifically cleaves DNA.

In this study, the sequence of DPV UL15 was optimized and ligated into the prokaryotic expression vector pET28a, and the soluble full-length UL15 protein was successfully purified. It was found that full-length DPV pUL15 functions as a metal-ion-dependent nuclease; its nuclease activity was significantly enhanced in the presence of Mn^2+^ or Mg^2+^. It was also found that the full-length DPV pUL15 can still cleave different DNA substrates, indicating that DPV pUL15 exhibits non-sequence-specific nuclease activity, which is similar to that of HSV-1 pUL15 and HCMV pUL89 [16,49]. Notably, unlike the truncated pUL15C, full-length pUL15 exhibited significantly enhanced DNA-binding ability at higher protein concentrations, a characteristic commonly observed in bacteriophage terminase large subunits [50,51]. This finding was consistent with structural analyses of HSV-1 pUL15, but represented the first experimental confirmation of this phenomenon. These results suggested that the N-terminal domain of pUL15 was primarily responsible for DNA binding, while the C-terminal domain exerted nuclease activity. The loss of nuclease function did not significantly affect DNA binding.

As one of the most powerful molecular motors identified, the terminase large subunit hydrolyzes ATP to drive the cleavage and packaging of concatemeric viral DNA. The ATPase activity of pUL15 in herpesviruses was first detected in HSV-1 in recent years, yet many aspects of its function remain unclear. In this study, we demonstrated that the N-terminus of DPV pUL15 could significantly hydrolyze ATP just like the full-length pUL15. The above results suggest that the N-terminal ATPase domain had the potential to bind to DNA, whereas the nuclease domain is dispensable for DNA binding. This study demonstrated that DPV pUL15 is a multifunctional enzyme, which not only possesses nuclease and ATPase activity, but also has DNA-binding ability.

The structural analysis of the HSV-1 terminase complex showed that the complex can be assembled into monomers, hexamers, and decadimers, and the hexamer is the predominant assembly form. In this ring-like structure, six copies of the ATPase domain are arranged inside the ring, forming a “funnel” channel structure. Six basic residues are observed inside this channel. These basic residues are likely to be responsible for binding to DNA during translocation [16,45]. A mechanism has also been reported in bacteriophage P74-26, in which the N-terminal domain of its large subunit TerL is responsible for gripping DNA to enable cleavage by the C-terminal domain [52]. DPV pUL15 hexamer, which was predicted by AlphaFold 3, displayed a similar structure with a channel that might be responsible for DNA binding and translocation. It was hypothesized that different enzymatic activities of DPV pUL15 played a synergistic role in the process of viral genome cleavage and packaging, such as performing actions like “DNA binding”–“hydrolyzing ATP”–“DNA translocation”, etc. and repeating these cycles until the complete genomic DNA was packaged into the capsid, and then it exerted its nuclease activity to complete the genome packaging.

DPV pUL15, as a multifunctional enzyme with independent domains, has direct implications for understanding viral pathogenesis in waterfowl. As the terminase complex is essential for the production of infectious virions, our findings suggest that the nuclease and ATPase activities of pUL15 are attractive targets for antiviral intervention. From a practical veterinary perspective, the results provide a foundation for several future research avenues: (i) high-throughput screening for compounds that disrupt UL15’s nuclease or ATPase activities; and (ii) engineering live-attenuated vaccines with mutations in the functional domain that impair viral replication. Furthermore, given the high conservation of the terminase complex across herpesviruses, the insights gained from this study not only contribute to the control of duck plague, but could also inform the development of broad-spectrum anti-herpesviral drugs targeting the terminase complex. Herpesviruses (such as HCMV, KSHV, and HSV) can cause a variety of serious diseases. Antiviral drugs currently used in clinical practice, such as acyclovir and ganciclovir, mainly play a role by inhibiting viral DNA polymerase [53,54]. However, the application of these drugs is often limited by adverse reactions and drug resistance mutations [55]. The concatemeric DNA packaging process is highly specific to herpesviruses and has no counterpart in host cellular processes, making it an ideal target for developing novel antiviral agents. Small molecule inhibitors targeting the nuclease or ATPase activities of the UL15 protein exhibit unique mechanisms of action and potentially improved safety profiles, offering new strategies for the prevention and treatment of herpesvirus infections. Therefore, targeting the terminase complex represents a promising direction for future antiviral drug development [56]. In this context, elucidating the structure and mechanism of the terminase complex and its role in DNA packaging will be of critical importance.

## 5. Conclusions

In summary, this study demonstrated that DPV pUL15 was a multifunctional enzyme with ATPase, nuclease, and DNA-binding activities. In vitro, DPV pUL15 did not specifically recognize or cleave DNA, its DNA-binding and ATP hydrolysis abilities were dominated by the N-terminus, and nuclease activity was carried out by the C-terminus. During viral replication, it was speculated that the different functions of DPV pUL15 worked in synergy to complete the cleavage and packaging of the viral genome. Our study provided clues for the future exploration of DPV genome cleavage and packaging. Meanwhile, it provided a basis for comparative analysis of the terminase complex of different hosts.

## Data Availability

All data presented in this study are included in the article/Supplementary Materials. Further inquiries can be directed to the corresponding author(s).

## Supplementary Materials

The following supporting information can be downloaded at https://www.mdpi.com/article/10.3390/vetsci12100992/s1: Figure S1: Purified DPV pUL15FL; Figure S2: Different substrates for the detection of pUL15FL nuclease activity; Figure S3: The effect of divalent metal ions on the nuclease activity of pUL15FL; Figure S4: Binding of pUL15FL to plasmid pUC118; Figure S5: Binding of pUL15FL to PAC and unPAC; Figure S6: Binding of pUL15FL to ssDNA; Figure S7: The effect of different divalent metal ions on the DNA-binding ability of pUL15FL; Figure S8: Purified soluble pUL15NM; Figure S9: Purified soluble pUL15N; Figure S10: Purified soluble pUL15C; Figure S11: Detection of the nuclease activity of pUL15 mutants; Figure S12: Detection of the DNA-binding ability of pUL15 mutants.

## Appendix A

**Table A1:** The primers used in this study.

Primers	Primer Sequences (5′ to 3′)
pET28a-UL15N-F/R	F: ATGGGTCGCGGATCCGAATTCATGTTCGGGGCAACTTTC
	R: GGTGGTGGTGGTGGTGCTCGAGTCTTTTCATTACCCGCTATCTCAC
pET28a-UL15NM-F/R	F: TCAACAAACGCGCAAGTATTT
	R: AAATACTTGCGCGTTTGTTGA
Pac-F/R	F: CCCCCCGCCAAAAAAGCC
	R: CGCCCTTCCATAGCAGTGCAT
unPac-F/R	F: TGCAGTGCTGCCATAACCATG
	R: GCTCCGGTTCCCAACGAT

**Table A2:** Sequences of DNA substrate used in the DNA binding assay.

Substrate	Substrate Sequences (5′ to 3′)
PAC	CCCCCCGCCAAAAAAGCCCCGCCCCCTTTTTTCGATGGCCCGGGAAATGGCAAAGGGGGTCCAACGCCTCTCCCGGCCGCGGCCGCGCAGAAAAAAATTTTTGCCTGACGTGCCTTCAATGCACTGCTATGGAAGGGCG
UnPac	TGCAGTGCTGCCATAACCATGAGTGATAACACTGCGGCCAACTTACTTCTGACAACGATCGGAGGACCGAAGGAGCTAACCGCTTTTTTGCACAACATGGGGGATCATGTAACTCGCCTTGATCGTTGGGAACCGGAGC
ssDNA	TCTCATCATTTTGGCAAAGAATTCATGGAGCAGAAGCTGATCTCAGAGGAGGACCTGGAGCAGAAGCTGATCTCAGAGGAGGACCTGGAGCAGAAGCTGATCTCAGAGGAGGACCTGTTCGGGGCAACTTTCGG

## References

[1] Baines J.D. (2011). Herpes simplex virus capsid assembly and DNA packaging: A present and future antiviral drug target. Trends Microbiol..

[2] Neuber S., Wagner K., Messerle M., Borst E.M. (2018). The C-terminal part of the human cytomegalovirus terminase subunit pUL51 is central for terminase complex assembly. J. Gen. Virol..

[3] Chiu S.H., Wu M.C., Wu C.C., Chen Y.C., Lin S.F., Hsu J.T., Yang C.S., Tsai C.H., Takada K., Chen M.R. (2014). Epstein-Barr virus BALF3 has nuclease activity and mediates mature virion production during the lytic cycle. J. Virol..

[4] Smits C., Chechik M., Kovalevskiy O.V., Shevtsov M.B., Foster A.W., Alonso J.C., Antson A.A. (2009). Structural basis for the nuclease activity of a bacteriophage large terminase. EMBO Rep..

[5] Zhao H., Christensen T.E., Kamau Y.N., Tang L. (2013). Structures of the phage Sf6 large terminase provide new insights into DNA translocation and cleavage. Proc. Natl. Acad. Sci. USA.

[6] McVoy M.A., Adler S.P. (1994). Human cytomegalovirus DNA replicates after early circularization by concatemer formation, and inversion occurs within the concatemer. J. Virol..

[7] Heming J.D., Huffman J.B., Jones L.M., Homa F.L. (2014). Isolation and characterization of the herpes simplex virus 1 terminase complex. J. Virol..

[8] Beard P.M., Duffy C., Baines J.D. (2004). Quantification of the DNA cleavage and packaging proteins U(L)15 and U(L)28 in A and B capsids of herpes simplex virus type 1. J. Virol..

[9] Deiss L.P., Chou J., Frenkel N. (1986). Functional domains within the a sequence involved in the cleavage-packaging of herpes simplex virus DNA. J. Virol..

[10] Heming J.D., Conway J.F., Homa F.L. (2017). Herpesvirus Capsid Assembly and DNA Packaging. Adv. Anat. Embryol. Cell Biol..

[11] Yang K., Homa F., Baines J.D. (2007). Putative terminase subunits of herpes simplex virus 1 form a complex in the cytoplasm and interact with portal protein in the nucleus. J. Virol..

[12] Huet A., Huffman J.B., Conway J.F., Homa F.L. (2020). Role of the Herpes Simplex Virus CVSC Proteins at the Capsid Portal Vertex. J. Virol..

[13] Beard P.M., Taus N.S., Baines J.D. (2002). DNA cleavage and packaging proteins encoded by genes U(L)28, U(L)15, and U(L)33 of herpes simplex virus type 1 form a complex in infected cells. J. Virol..

[14] Neuber S., Wagner K., Goldner T., Lischka P., Steinbrueck L., Messerle M., Borst E.M. (2017). Mutual Interplay between the Human Cytomegalovirus Terminase Subunits pUL51, pUL56, and pUL89 Promotes Terminase Complex Formation. J. Virol..

[15] Abbotts A.P., Preston V.G., Hughes M., Patel A.H., Stow N.D. (2000). Interaction of the herpes simplex virus type 1 packaging protein UL15 with full-length and deleted forms of the UL28 protein. J. Gen. Virol..

[16] Yang Y., Yang P., Wang N., Chen Z., Su D., Zhou Z., Rao Z., Wang X. (2020). Architecture of the herpesvirus genomepackaging complex and implications for DNA translocation. Protein Cell.

[17] Mahler B.P., Bujalowski P.J., Mao H., Dill E.A., Jardine P.J., Choi K.H., Morais M.C. (2020). NMR structure of a vestigial nuclease provides insight into the evolution of functional transitions in viral dsDNA packaging motors. Nucleic Acids Res..

[18] Yang K., Wills E.G., Baines J.D. (2011). A mutation in UL15 of herpes simplex virus 1 that reduces packaging of cleaved genomes. J. Virol..

[19] White C.A., Stow N.D., Patel A.H., Hughes M., Preston V.G. (2003). Herpes simplex virus type 1 portal protein UL6 interacts with the putative terminase subunits UL15 and UL28. J. Virol..

[20] Yang K., Wills E., Baines J.D. (2009). The putative leucine zipper of the UL6-encoded portal protein of herpes simplex virus 1 is necessary for interaction with pUL15 and pUL28 and their association with capsids. J. Virol..

[21] Trus B.L., Cheng N., Newcomb W.W., Homa F.L., Brown J.C., Steven A.C. (2004). Structure and polymorphism of the UL6 portal protein of herpes simplex virus type 1. J. Virol..

[22] Hwang J.S., Bogner E. (2002). ATPase activity of the terminase subunit pUL56 of human cytomegalovirus. J. Biol. Chem..

[23] Salmon B., Cunningham C., Davison A.J., Harris W.J., Baines J.D. (1998). The herpes simplex virus type 1 U(L)17 gene encodes virion tegument proteins that are required for cleavage and packaging of viral DNA. J. Virol..

[24] McNab A.R., Desai P., Person S., Roof L.L., Thomsen D.R., Newcomb W.W., Brown J.C., Homa F.L. (1998). The product of the herpes simplex virus type 1 UL25 gene is required for encapsidation but not for cleavage of replicated viral DNA. J. Virol..

[25] Lamberti C., Weller S.K. (1998). The herpes simplex virus type 1 cleavage/packaging protein, UL32, is involved in efficient localization of capsids to replication compartments. J. Virol..

[26] Ogasawara M., Suzutani T., Yoshida I., Azuma M. (2001). Role of the UL25 gene product in packaging DNA into the herpes simplex virus capsid: Location of UL25 product in the capsid and demonstration that it binds DNA. J. Virol..

[27] Gates S.N., Martin A. (2020). Stairway to translocation: AAA+ motor structures reveal the mechanisms of ATP-dependent substrate translocation. Protein Sci..

[28] Puchades C., Sandate C.R., Lander G.C. (2020). The molecular principles governing the activity and functional diversity of AAA+ proteins. Nat. Rev. Mol. Cell Biol..

[29] Iyer L.M., Makarova K.S., Koonin E.V., Aravind L. (2004). Comparative genomics of the FtsK-HerA superfamily of pumping ATPases: Implications for the origins of chromosome segregation, cell division and viral capsid packaging. Nucleic Acids Res..

[30] Selvarajan Sigamani S., Zhao H., Kamau Y.N., Baines J.D., Tang L. (2013). The structure of the herpes simplex virus DNA-packaging terminase pUL15 nuclease domain suggests an evolutionary lineage among eukaryotic and prokaryotic viruses. J. Virol..

[31] Scheffczik H., Savva C.G., Holzenburg A., Kolesnikova L., Bogner E. (2002). The terminase subunits pUL56 and pUL89 of human cytomegalovirus are DNA-metabolizing proteins with toroidal structure. Nucleic Acids Res..

[32] Theiß J., Sung M.W., Holzenburg A., Bogner E. (2019). Full-length human cytomegalovirus terminase pUL89 adopts a two-domain structure specific for DNA packaging. PLoS Pathog..

[33] Yang Q., Liu Y., Wang M., Wu Y., Bin T., Ou X., Mao S., Huang J., Sun D., Gao Q. (2023). Duck plague virus pUL15 performs a nonspecial cleavage activity through its C terminal nuclease domain in vitro. Vet. Microbiol..

[34] Adelman K., Salmon B., Baines J.D. (2001). Herpes simplex virus DNA packaging sequences adopt novel structures that are specifically recognized by a component of the cleavage and packaging machinery. Proc. Natl. Acad. Sci. USA.

[35] Wu H., Sampson L., Parr R., Casjens S. (2002). The DNA site utilized by bacteriophage P22 for initiation of DNA packaging. Mol. Microbiol..

[36] Chai S., Lurz R., Alonso J.C. (1995). The small subunit of the terminase enzyme of Bacillus subtilis bacteriophage SPP1 forms a specialized nucleoprotein complex with the packaging initiation region. J. Mol. Biol..

[37] Bogner E., Radsak K., Stinski M.F. (1998). The gene product of human cytomegalovirus open reading frame UL56 binds the pac motif and has specific nuclease activity. J. Virol..

[38] Roy A., Bhardwaj A., Datta P., Lander G.C., Cingolani G. (2012). Small terminase couples viral DNA binding to genome-packaging ATPase activity. Structure.

[39] Dhama K., Kumar N., Saminathan M., Tiwari R., Karthik K., Kumar M.A., Palanivelu M., Shabbir M.Z., Malik Y.S., Singh R.K. (2017). Duck virus enteritis (duck plague)—A comprehensive update. Vet. Q..

[40] Camacho A.G., Gual A., Lurz R., Tavares P., Alonso J.C. (2003). Bacillus subtilis bacteriophage SPP1 DNA packaging motor requires terminase and portal proteins. J. Biol. Chem..

[41] Weitao T., Grandinetti G., Guo P. (2023). Revolving ATPase motors as asymmetrical hexamers in translocating lengthy dsDNA via conformational changes and electrostatic interactions in phi29, T7, herpesvirus, mimivirus, E. coli, and Streptomyces. Exploration.

[42] Homa F.L., Brown J.C. (1997). Capsid assembly and DNA packaging in herpes simplex virus. Rev. Med. Virol..

[43] Miller J.T., Zhao H., Masaoka T., Varnado B., Cornejo Castro E.M., Marshall V.A., Kouhestani K., Lynn A.Y., Aron K.E., Xia A. (2018). Sensitivity of the C-Terminal Nuclease Domain of Kaposi’s Sarcoma-Associated Herpesvirus ORF29 to Two Classes of Active-Site Ligands. Antimicrob. Agents Chemother..

[44] Sheaffer A.K., Newcomb W.W., Gao M., Yu D., Weller S.K., Brown J.C., Tenney D.J. (2001). Herpes simplex virus DNA cleavage and packaging proteins associate with the procapsid prior to its maturation. J. Virol..

[45] Hilbert B.J., Hayes J.A., Stone N.P., Duffy C.M., Sankaran B., Kelch B.A. (2015). Structure and mechanism of the ATPase that powers viral genome packaging. Proc. Natl. Acad. Sci. USA.

[46] Xu R.G., Jenkins H.T., Chechik M., Blagova E.V., Lopatina A., Klimuk E., Minakhin L., Severinov K., Greive S.J., Antson A.A. (2017). Viral genome packaging terminase cleaves DNA using the canonical RuvC-like two-metal catalysis mechanism. Nucleic Acids Res..

[47] Yang W. (2011). Nucleases: Diversity of structure, function and mechanism. Q. Rev. Biophys..

[48] Alam T.I., Draper B., Kondabagil K., Rentas F.J., Ghosh-Kumar M., Sun S., Rossmann M.G., Rao V.B. (2008). The headful packaging nuclease of bacteriophage T4. Mol. Microbiol..

[49] Nadal M., Mas P.J., Blanco A.G., Arnan C., Solà M., Hart D.J., Coll M. (2010). Structure and inhibition of herpesvirus DNA packaging terminase nuclease domain. Proc. Natl. Acad. Sci. USA.

[50] Alam T.I., Rao V.B. (2008). The ATPase domain of the large terminase protein, gp17, from bacteriophage T4 binds DNA: Implications to the DNA packaging mechanism. J. Mol. Biol..

[51] Schwartz C., De Donatis G.M., Fang H., Guo P. (2013). The ATPase of the phi29 DNA packaging motor is a member of the hexameric AAA+ superfamily. Virology.

[52] Hilbert B.J., Hayes J.A., Stone N.P., Xu R.G., Kelch B.A. (2017). The large terminase DNA packaging motor grips DNA with its ATPase domain for cleavage by the flexible nuclease domain. Nucleic Acids Res..

[53] Biron K.K. (2006). Antiviral drugs for cytomegalovirus diseases. Antiviral Res..

[54] Elion G.B. (1993). Acyclovir: Discovery, mechanism of action, and selectivity. J. Med. Virol..

[55] Muller C., Alain S., Hantz S. (2023). Identification of a leucine-zipper motif in pUL51 essential for HCMV replication and potential target for antiviral development. Antiviral Res..

[56] Hakki M. (2020). Moving Past Ganciclovir and Foscarnet: Advances in CMV Therapy. Curr. Hematol. Malig. Rep..

