# Supplementary material for: Duck Plague Virus Full-Length UL15 Protein Is a Multifunctional Enzyme Which Not Only Possesses Nuclease Activity but Also Exerts ATPase and DNA-Binding Activity
Source: Vet Sci. 2025 Oct 14;12(10):992. doi: 10.3390/vetsci12100992 (PMC12567909; doi:10.3390/vetsci12100992)
Supplement: Supplementary file 1 [file vetsci-12-00992-s001.zip › vetsci-3888413-supplementary.pdf]

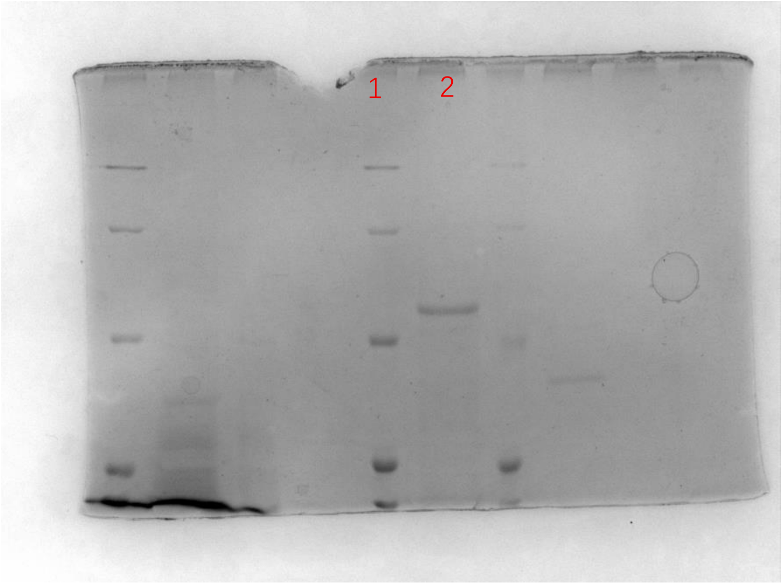

Figure S1. Purified DPV pUL15FL. Lane 1: Protein Marker; Lane 2: Purified pUL15FL.

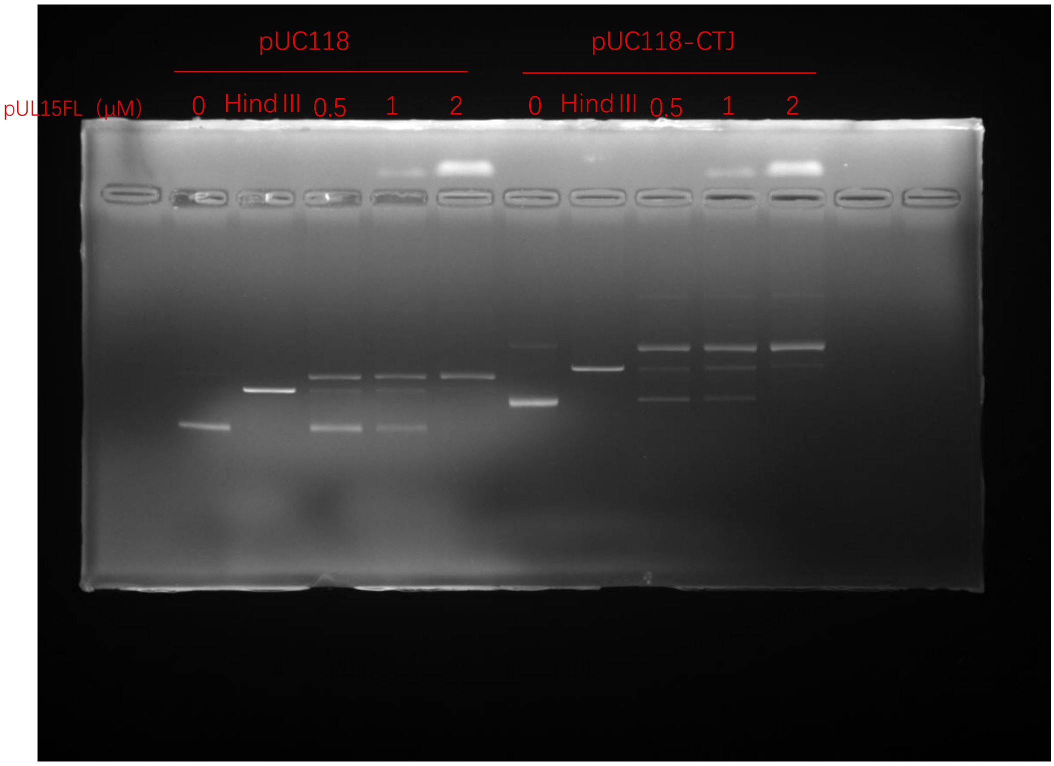

Figure S2. Different substrates for the detection of pUL15FL nuclease activity. pUC118 is a vector plasmid, and pUC118-CTJ contains the DPV genome concatemeric terminus junction (CTJ).

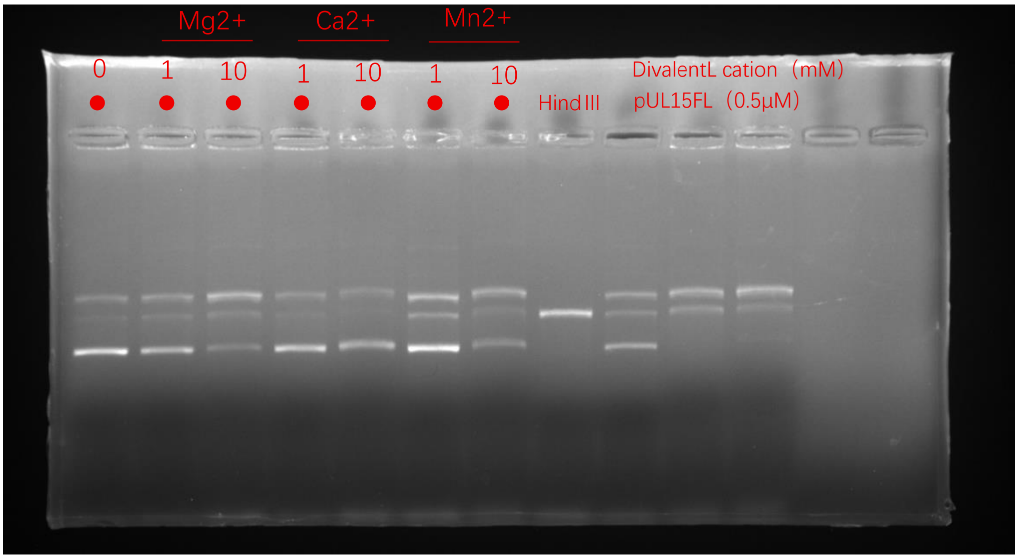

Figure S3. The effect of divalent metal ions on the nuclease activity of pUL15FL.

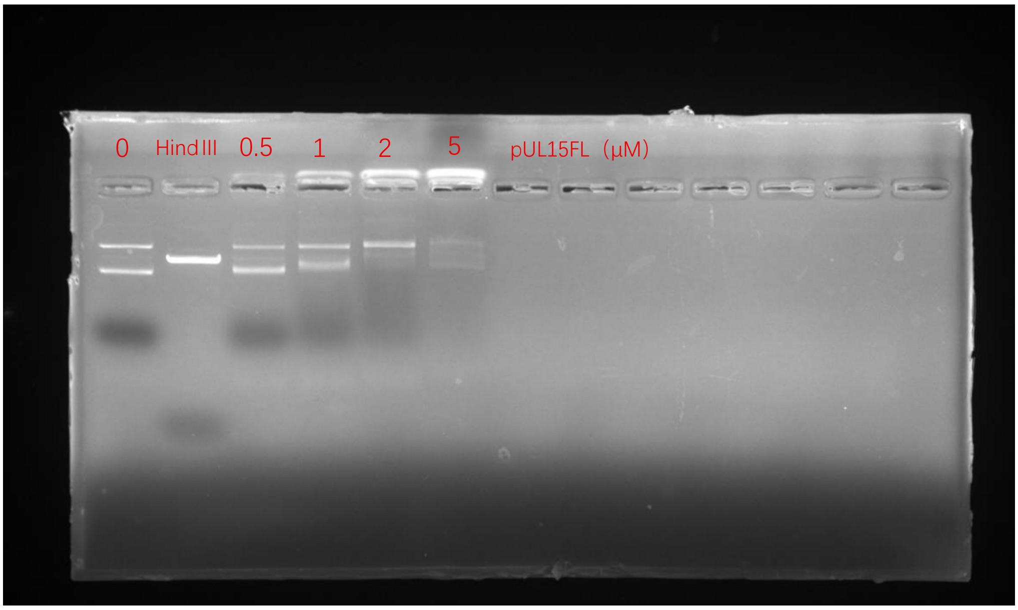

Figure S4. Binding of pUL15FL to plasmid pUC118.

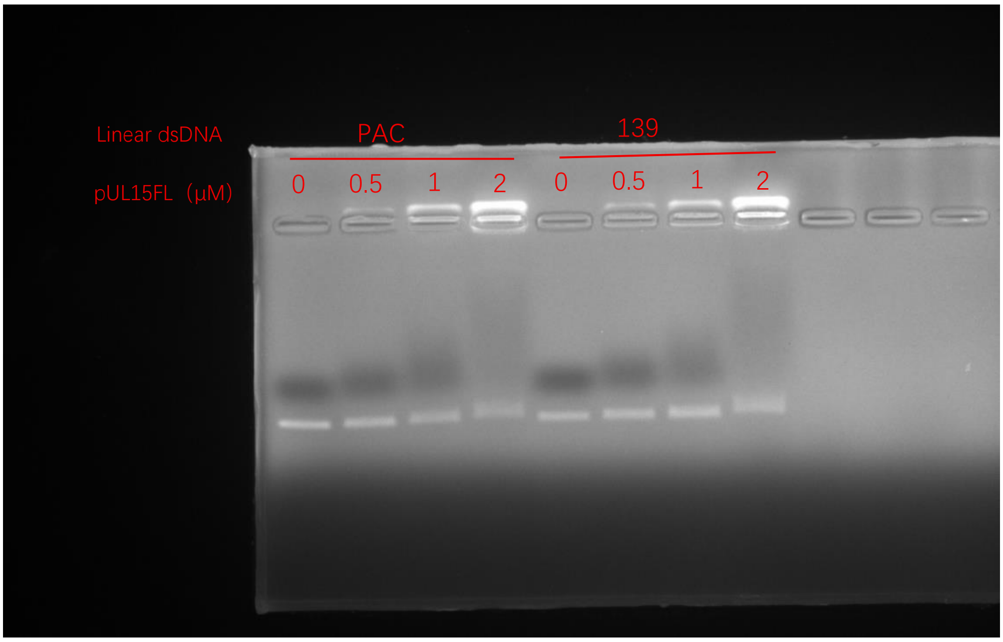

Figure S5. Binding of pUL15FL to PAC and unPAC.

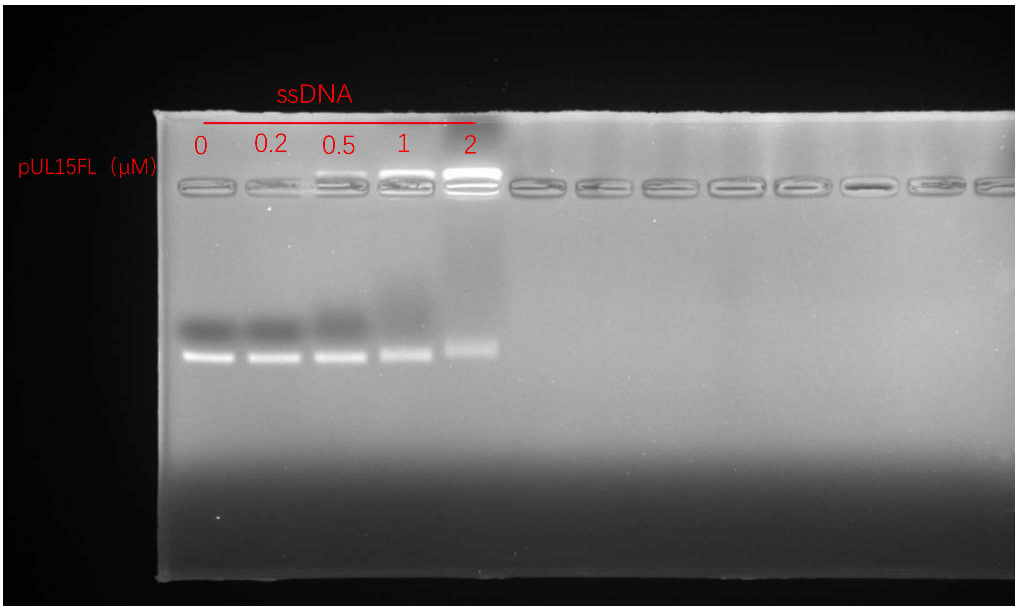

Figure S6. Binding of pUL15FL to ssDNA.

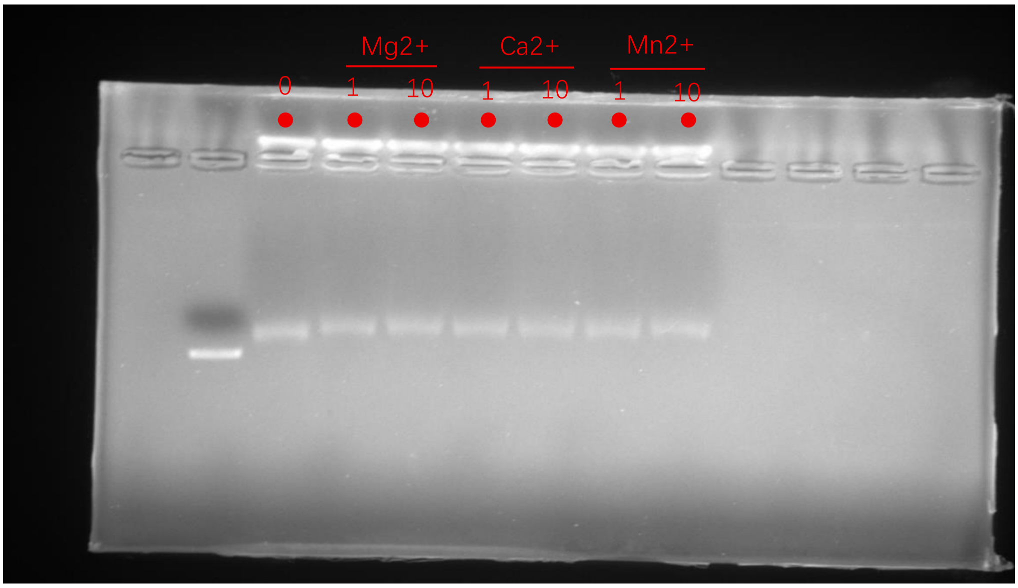

Figure S7. The effect of different divalent metal ions on the DNA-binding ability of pUL15FL.

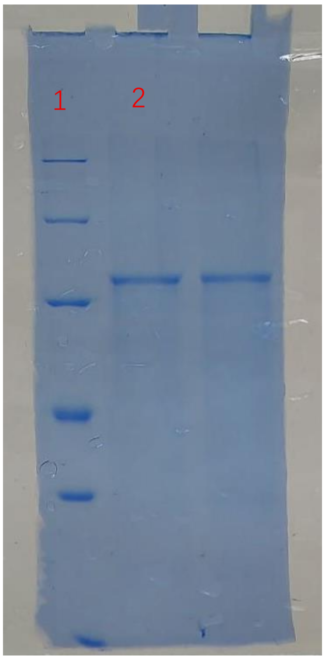

Figure S8. Purified soluble pUL15NM. Lane 1: Protein Marker; Lane 2: Purified pUL15NM.

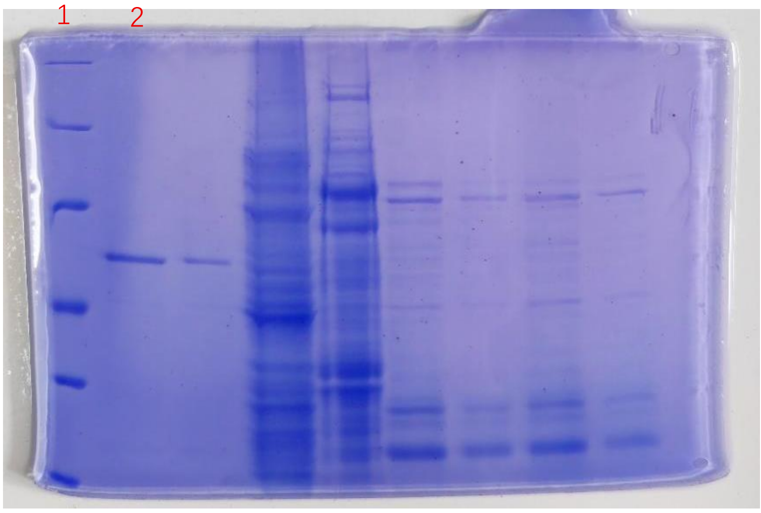

Figure S9. Purified soluble pUL15N. Lane 1: Protein Marker; Lane 2: Purified pUL15N.

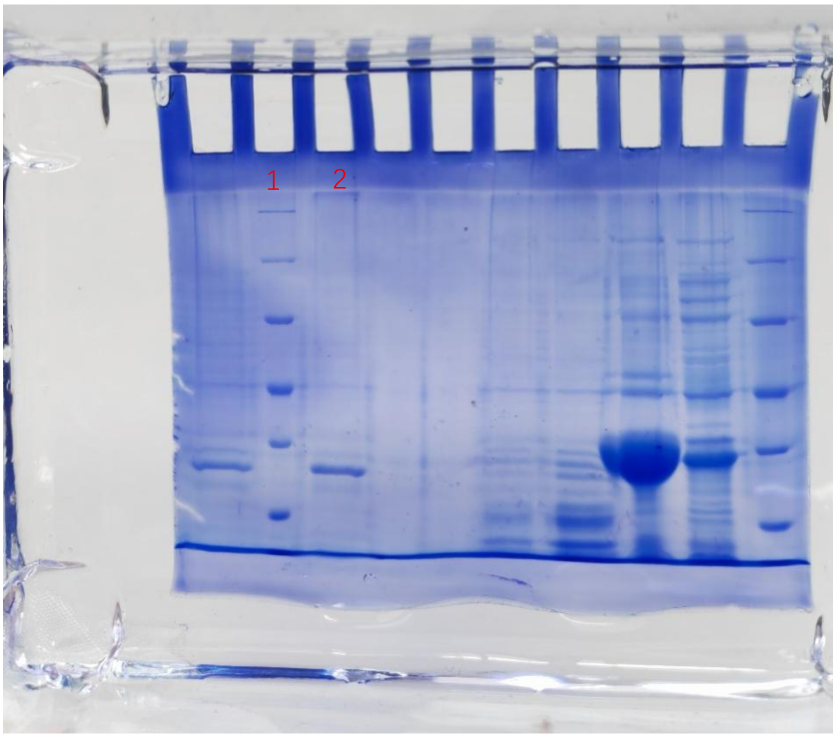

Figure S10. Purified soluble pUL15C. Lane 1: Protein Marker; Lane 2: Purified pUL15C.

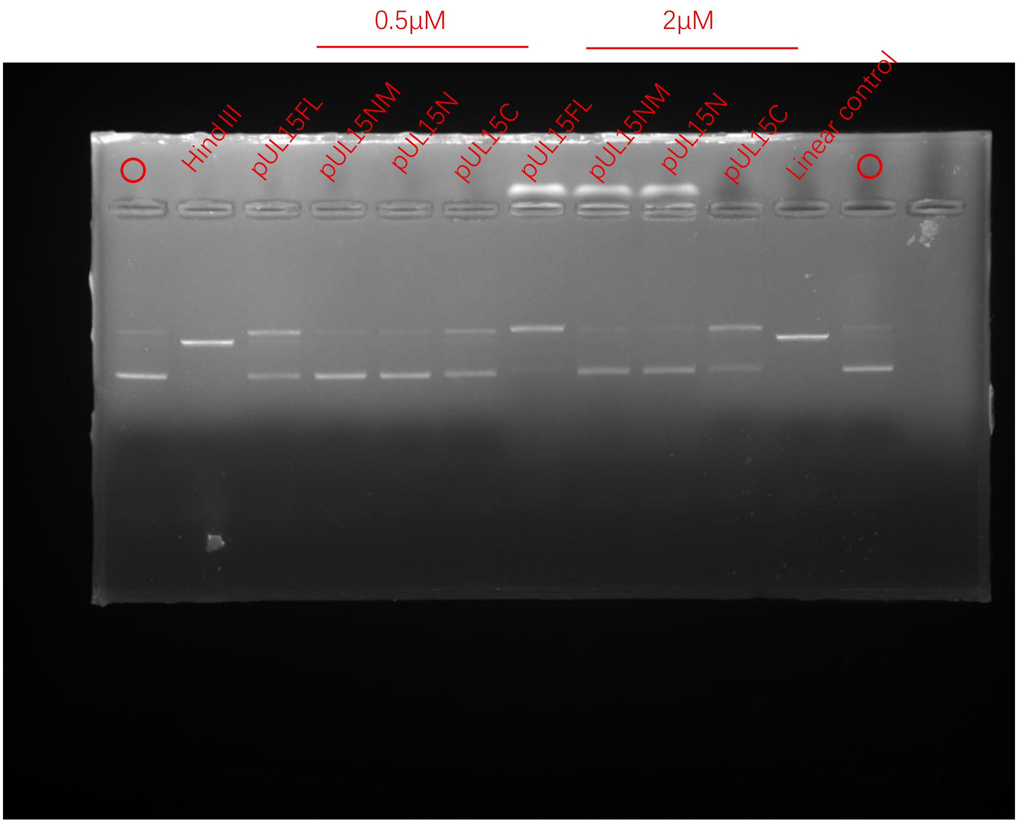

Figure S11. Detection of the nuclease activity of pUL15 mutants. Plasmid pUC118 is used as the substrate, and the concentration of each protein is 0.5 µM and 2 µM.

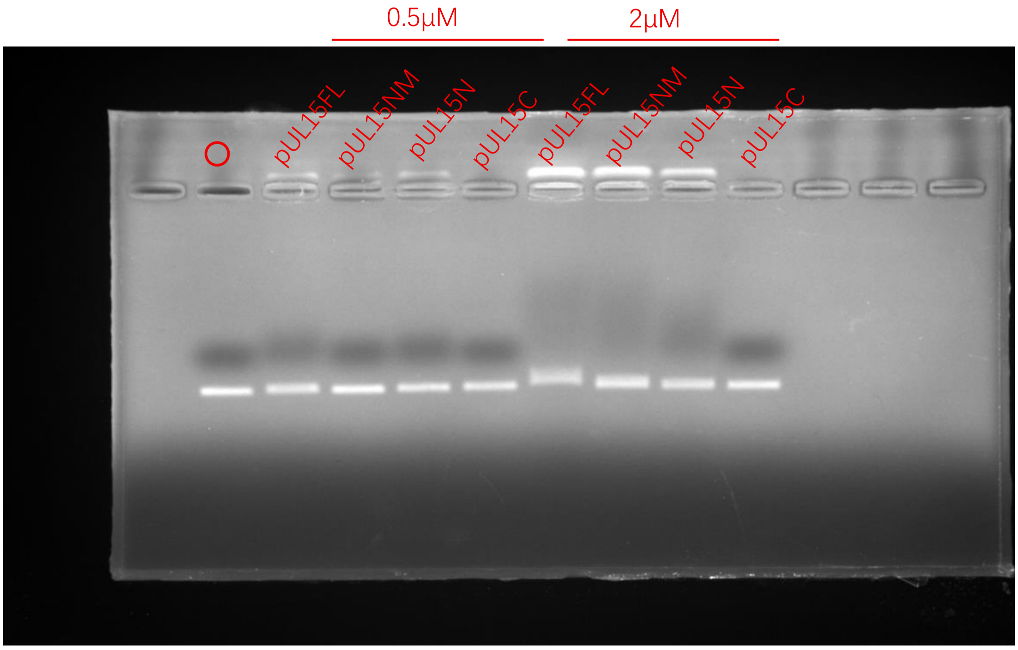

Figure S12. Detection of the DNA-binding ability of pUL15 mutants. dsDNA, PAC, is used as the substrate, and the concentration of each protein is 0.5 µM and 2 µM.
